# *Cryptosporidium parvum* infection alters the intestinal mucosa transcriptome in neonatal calves: implications for immune function

**DOI:** 10.3389/fimmu.2024.1351427

**Published:** 2024-01-22

**Authors:** Arash Veshkini, Franziska Dengler, Lisa Bachmann, Wendy Liermann, Christiane Helm, Reiner Ulrich, Cora Delling, Christa Kühn, Harald M. Hammon

**Affiliations:** ^1^Research Institute for Farm Animal Biology, Institute of Nutritional Physiology “Oskar Kellner”, Dummerstorf, Germany; ^2^Institute of Physiology, Pathophysiology and Biophysics, University of Veterinary Medicine, Vienna, Austria; ^3^Faculty of Agriculture and Food Science, University of Applied Science Neubrandenburg, Neubrandenburg, Germany; ^4^Institutue for Veterinary Pathology, Leipzig University, Leipzig, Germany; ^5^Institute of Veterinary Parasitology, Leipzig University, Leipzig, Germany; ^6^Research Institute for Farm Animal Biology, Institute of Genome Biology, Dummerstorf, Germany; ^7^Agricultural and Environmental Faculty, University Rostock, Rostock, Germany

**Keywords:** innate immunity, diarrhea, next generation sequencing, adaptive immunity, chemotaxis, bovine, protozoa, parasite-host interaction

## Abstract

One of the leading causes of infectious diarrhea in newborn calves is the apicomplexan protozoan *Cryptosporidium parvum* (*C. parvum*). However, little is known about its immunopathogenesis. Using next generation sequencing, this study investigated the immune transcriptional response to *C. parvum* infection in neonatal calves. Neonatal male Holstein-Friesian calves were either orally infected (N = 5) or not (CTRL group, N = 5) with *C. parvum* oocysts (gp60 subtype IIaA15G2R1) at day 1 of life and slaughtered on day 7 after infection. Total RNA was extracted from the jejunal mucosa for short read. Differentially expressed genes (DEGs) between infected and CTRL groups were assessed using DESeq2 at a false discovery rate < 0.05. Infection did not affect plasma immunohematological parameters, including neutrophil, lymphocyte, monocyte, leucocyte, thrombocyte, and erythrocyte counts as well as hematocrit and hemoglobin concentration on day 7 post infection. The immune-related DEGs were selected according to the UniProt immune system process database and were used for gene ontology (GO) and pathway enrichment analysis using Cytoscape (v3.9.1). Based on GO analysis, DEGs annotated to mucosal immunity, recognizing and presenting antigens, chemotaxis of neutrophils, eosinophils, natural killer cells, B and T cells mediated by signaling pathways including toll like receptors, interleukins, tumor necrosis factor, T cell receptor, and NF-KB were upregulated, while markers of macrophages chemotaxis and cytosolic pattern recognition were downregulated. This study provides a holistic snapshot of immune-related pathways induced by *C. parvum* in calves, including novel and detailed feedback and feedforward regulatory mechanisms establishing the crosstalk between innate and adaptive immune response in neonate calves, which could be utilized further to develop new therapeutic strategies.

## 1 Introduction

Infectious neonatal (< 1 month old) calf diarrhea (NCD) is a serious worldwide health issue with a high prevalence rate (18.5% of calf disorders in Germany), resulting in significant economic losses mainly related to reduced weight gain and palliative treatments (1). According to veterinary surveillance reports from the UK and Germany, the protozoan parasite *Cryptosporidium* was the most commonly diagnosed cause of NCD in fecal samples submitted to laboratories or taken postmortem (2, 3). *C. parvum* is host-adapted to cattle and associated with clinical disease including acute to chronic catarrhal enteritis in neonatal calves that mainly begins in the ileum but is also observed in the distal jejunum (3).

*C. parvum* sporozoites primarily invade the ileal mucosa, specifically the epithelial cells at the ileocecal junction, where they complete their life cycle (3). Released merozoites re-infect the host by their invasion of neighboring epithelial cells and infectious oocysts are shed into the environment (4). As a result of infection, an innate inflammatory response is initiated in epithelial cells by ligation of Toll-like receptors (TLRs) that coalesce around parasites, activating the nuclear factor-kappa B (NF-κB) signaling pathway (5). Consequently, the transcription factor NF-κB initiates a signal transduction that regulates the expression of cytokines, chemokines, interferon regulatory factors (IRFs), and antimicrobial peptides (6). The innate immune response against *C. parvum* involving the recruitment of natural killer (NK) cells, dendritic cells (DCs), macrophages, and neutrophils was recently reviewed by (7). However, to fully eradicate *C. parvum*, the adaptive immune response including T cells and interferon (IFN) is often required (8).

With considerable amounts of lymphoid tissue and a large population of scattered innate and adaptive hematopoietic cells (embedded within the sub-mucosa), the intestine is a critical organ of the immune system (9, 10). In the jejunum and ileum of calves, Peyer’s patches (PPs) serve as immune sensors and as lymphoid organs responsible for the generation and development of B cells (11). PPs form follicles, which primarily consist of B cells, but also contain T cells, plasma cells, mast cells, eosinophils, macrophages, and basophils (12). Calves are born with all essential components of the adaptive immune response, albeit immature. For instance, T cell subsets including CD4+ and CD8+ do not reach peak levels until 8 months of life (13). Endogenous IgM is produced only after 4 days of life and reaches functional levels only after 8 days (14). For immunoglobulin (Ig)A, IgG1, and IgG2, it takes 16 to 32 days to reach functional levels (15). Consequently, newborn calves are primarily protected by their innate immune system and their responses to antigens are weaker, slower, and less specific than in adults (13).

It has been shown that *C. parvum* infection results in partial enlargement and swelling of the host mesenteric lymph nodes, accompanied by a strong infiltration of mononuclear cells and neutrophil granulocytes in the lamina propria of the mucosa (16, 17). Despite the number of studies having investigated immunity against *C. parvum* infections in human and animal models, the immunopathogenesis in calves is still poorly understood and inter-species differences hinder the development of vaccines. Therefore, a comprehensive understanding of host–pathogen interactions on calves’ physiological functions is urgently needed to develop future interventions. In this *in vivo* study, next-generation sequencing (NGS) was used to explore the calf immune response to *C. parvum* by targeting the jejunum mucosa, one of the main sites of invasion.

## 2 Material and methods

### 2.1 Animals and experimental design

As previously published in detail by Dengler et al. (18), the experimental design complied with German animal protection legislation and was licensed by the Landesdirektion Leipzig, Germany as TVV 19/20.

Briefly, the trial was conducted at the University of Leipzig with 10 newborn male Holstein-Friesian calves, who were fed 3 L of pooled colostrum after birth (= day 1 of life). Two separate barns (CTRL and infected) were used to stall the calves; the infected group was administered 2*107 *C. parvum* oocysts orally (n = 5, body weight = 41.7 ± 5.1 kg), while the CTRL group (n = 5, body weight = 44.6 ± 3.3 kg) received pure water. The dose of infection was determined based on the protocol for passaging the in-house-strain at the Institute of Parasitology, Leipzig University (19). The day of infection was considered as day 1 of the study. Calves were fed 3 x 2 L milk replacer daily, plus 2 L electrolytes daily from day 4 following treatment and slaughtered at day 7 after infection. Day 7 was selected for further measurements because preliminary clinical observations and measurements of the oocyst shedding indicated that the peak of infection occurred between day 4-7 in calves. However, in this experiment, the peak of infection occurred earlier than day 7.

Calves were housed on wood shavings and had free access to water. During the post-infection period, the calves were monitored clinically, including the diagnosis of diarrhea by assessing fecal consistency and quantifying fecal shedding of *C. parvum* oocysts using the commercial immunofluorescence assay kit MERIFLUOR® Cryptosporidium/Giardia (Meridian Bioscience, Inc., Cincinnati, USA).

### 2.2 Blood sampling and immunohematological analyses

Blood samples were collected on day 7 of infection to measure immunohematological parameters. Blood samples were drawn from the jugular vein into evacuated tubes (Vacuette, Greiner Bio-One International AG, Kremsmünster, Austria) coated with lithium-heparin (12-30 IU heparin per 10 mL) and hematocrit (HCT), hemoglobin (HGB), erythrocyte mean corpuscular volume (MCV), erythrocyte mean corpuscular hemoglobin (MCH), erythrocyte mean corpuscular hemoglobin concentration (MCHC) as well as immune cell counts were measured in the central laboratory of the large animal clinics of the University of Leipzig with an Advia 120 Hematology System (Siemens, Erlangen, Germany).

### 2.3 Intestinal mucosa sampling

Calves were slaughtered on day 7 of infection and the mucosa was manually stripped off from the underlying muscle. The extracted tissues were snap frozen in liquid nitrogen and stored at -80°C until further analysis.

### 2.4 RNA extraction and library preparation

RNA was isolated from grinded frozen tissue via the NucleoSpin RNA kit (Macherey-Nagel, Düren, Germany). Assessment of RNA integrity was conducted via Bioanalyzer (Agilent Genomics, Waldbronn, Germany) and quantification of RNA was performed by Qbit (Fisher Scientific). The isolated RNA was tested for DNA contamination with an established PCR test described by Weikard et al. (20). Libraries for mRNA transcriptome sequencing were prepared with an input of 1 microgram total RNA for the Illumina TruSeq Stranded mRNA library preparation kit (Illumina, San Diego, USA) After quality control on a Bioanalyzer (Agilent Genomics, Waldbronn, Germany), the libraries were sequenced on an HiSeq2500 (Illumina, San Diego, USA) with 2 x 100 bp cycles.

### 2.5 Read mapping and differential expression analysis

After demultiplexing, the obtained reads were analyzed via the nf-core RNAseq v3.4 pipeline https://nf-co.re/rnaseq/3.12.0) with default settings. The software versions included are listed in **Supplementary Table** ck1 (Software_versions_nfcore_RNAseq_3_4.docx). The nf-core standard enables full reproducibility all steps including quality control of reads, read trimming, alignment and expression counts at gene and transcript level. For read alignment, the ARS-UCD1.2_Btau5.0.1Y assembly run 9 (21) without unplaced NKL contigs served as backbone. The *Bos taurus* Ensembl genome annotation v105 provided gene and transcript coordinates.

Differential expression analysis was conducted via DESeq2 1.26.0 (22). Only transcripts with a TPM value > 1 in at least 4 samples were kept for further analysis to avoid spurious results. Input for the statistical tests were gene expression counts as calculated via Salmon (v. 1.5.2) within the nf-core RNAseq pipeline. The model for establishing differential expression included the effect of control group versus infection. P-values from the initial test were adjusted via Benjamini-Hochberg to account for multiple testing. Only genes with an FDR_BH_ < 0.05 were accepted as statistically significant.

### 2.6 Statistical and bioinformatics analysis

Immunohematological parameters were first tested for normality using the Kolmogorov-Smirnov test in SAS (v8.2) and log transformed. Differences between groups were analyzed using an unpaired Student’s two-tailed t-test with a 95% confidence interval in SAS. Data are presented as means ± SD and P values < 0.05 were considered significant.

To identify a subset of differentially expressed genes (DEGs) involved in immunity pathways, DEGs were first searched against the UniProt immune system process database (02.2023), and mapped DEGs were extracted and named as immune-related DEGs. Moderate to strong correlations were observed between the relative expression of (selected) immune-related DEGs and their respective proteome abundance (LC-MS/MS, **Supplementary Figure S1**). A gene–gene interaction network of DEGs was constructed to investigate the interactions of immune-related DEGs. The network nodes and edges data were retrieved from the STRING plugin and plotted using yFiles radial layout in Cytoscape software (v3.9.1).

Gene ontology (GO) annotation and functional enrichment analysis including biological process, cellular component, and Kyoto Encyclopedia of Genes and Genomes (KEGG) pathways was executed by ClueGo (v2.5.9) with Cytoscape software. False discovery rate (FDR) < 0.05 and having at least two identified DEG within each pathway were set as a threshold to identify significantly enriched GO terms and KEGG pathways. For grouping we set the parameters to clustering based on Kappa score. CluePedia (v1.5.10) plugin was used to highlight genes which were initial for pathway enrichment or shared between pathways.

## 3 Results

### 3.1 Calves’ zootechnical performance, immunohematology and *C. parvum* diagnosis

**Figure 1:**
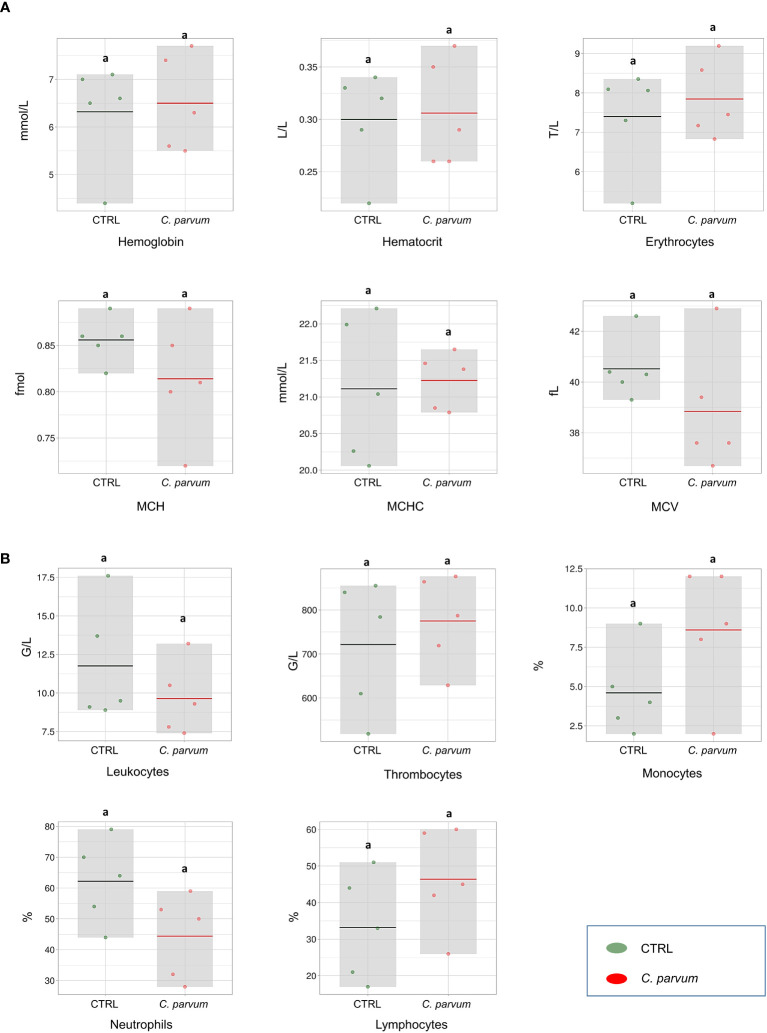
Blood hematological measurements **(A)** and immune cell counts **(B)** of calves infected or not with *Cryptosporidium parvum (C. parvum)* represented as box-plots (mean and standard deviation). The dots represent individual samples, and the colored line in the box represents the mean. MCH, mean corpuscular hemoglobin; MCHC, mean corpuscular hemoglobin concentration; MCV, mean corpuscular volume.

In our previous report of this study (18), the calves’ clinical data and zootechnical performance were provided in detail, also the presence of *C. parvum* oocysts (sporozoites) on the luminal border of calves’ ileum were confirmed by immunostaining. The hematological parameters as well as immune cell counts were evaluated in the calves infected or not with *C. parvum* on day 7 of infection. None of the hematology parameters comprising HBG, HCT, erythrocytes, MCH, MCHC, and MCV were significantly affected by the infection (**Figure 1A**). Also, we observed no significant differences in neutrophil, lymphocyte, monocyte, leucocyte, and thrombocyte counts between treatment groups on day 7 post infection (**Figure 1B**).

### 3.2 Overview of identified genes and their major patterns within calves’ intestinal mucosa

In total, 12,908 gene sequences were identified in mucosal cells at a threshold of transcripts per million (TPM) > 1 in at least four samples, in which 11,844 were known genes and 1,064 were novel genes (**Supplementary Table S1**). Novel genes were excluded from the analysis. Beside the protein coding genes, there were 145 long non-coding RNAs (lncRNAs), 9 microRNAs, 7 ribonucleic acids (rRNA) and ribozymes (ribonucleic acid enzymes), and 85 small nucleolar RNAs (snoRNAs and snRNAs) (**Supplementary S1**). There is increasing evidence that lncRNAs, such as U90926 (23), NR_045064 (24), and NR_126553 (25), play critical roles in host-pathogen interactions and signal transduction during *C. parvum* induced diarrhea. Accordingly, we found eight differentially expressed lncRNAs in infected group which are not yet categorized and thus were not included in the final list, but might be interesting for further investigation (**Supplementary S2**).

**Figure 2:**
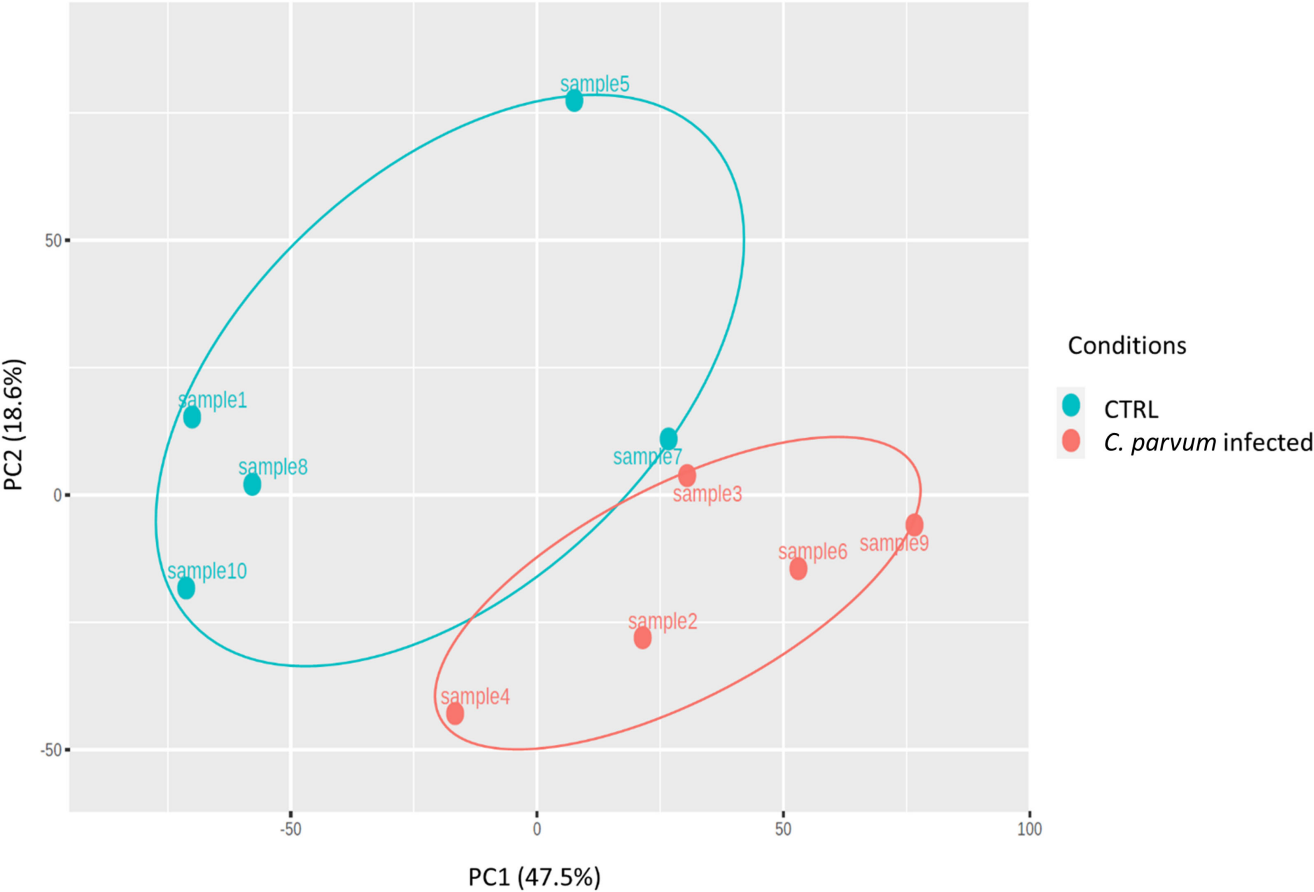
Principal component analysis (PCA) was performed on transformed gene expression data from calves’ jejunum mucosa infected or not with *Cryptosporidium parvum (C. parvum).* PCA identified two distinct clusters in the data separated along the first principal component associated with the *C. parvum* infectious. The percentage of variance accounted for by each PC is included in brackets.

The relative similarities of gene expression patterns in the mucosal cells of infected and non-infected calves were evaluated by an unsupervised principal component analysis (PCA). **Figure 2** represents the PCA scatterplot of the first two principal components based on transcriptome profiles of infected and non-infected calves. PC1 and PC2 explain 47.5% and 18.6% of the total variation, respectively. The infected calves formed a distinct cluster, indicating large differences in gene expression between the two groups. The largest distance between infected and non-infected calves was found in the first component (PC1).

### 3.3 Differential gene expression between infected and non-infected calves and their gene ontology analysis

**Figure 3:**
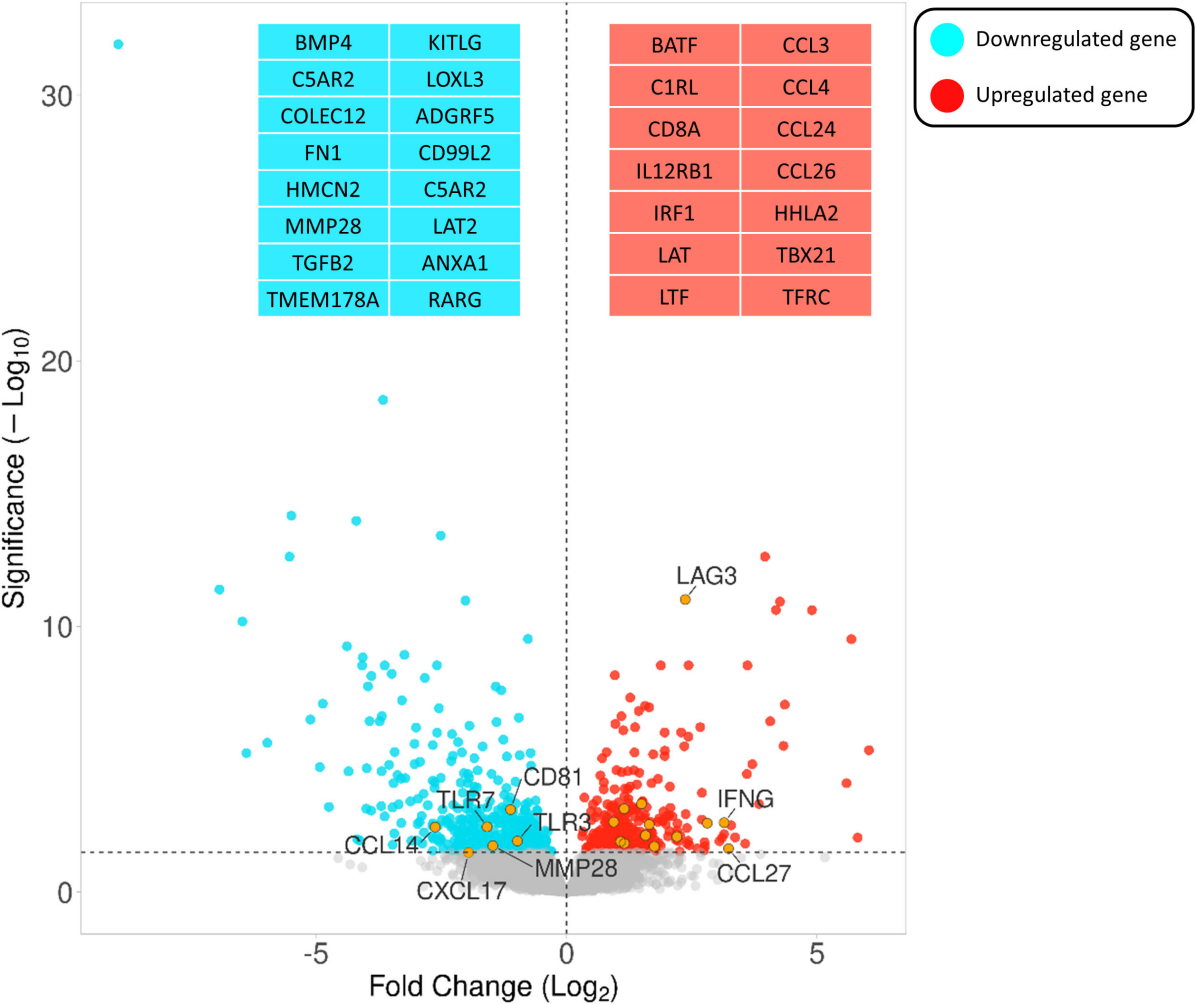
Volcano plot highlighting the gene expression differences between infected vs non-infected calves based on FDR < 0.05. The Y- and X-axes represent –log10 (P-value) and the log 2-based fold change of the expression data, respectively. Blue and red dots represent genes that are down- and upregulated, respectively. Orange circles indicate the positions of a few immune-associated genes among all differentially expressed genes.

Comparing the infected and non-infected group (with a FDR cut-off value < 0.05), 562 genes were downregulated and 405 were upregulated (**Supplementary S2**). **Figure 3** depicts a volcano plot of the DEGs.

**Figure 4:**
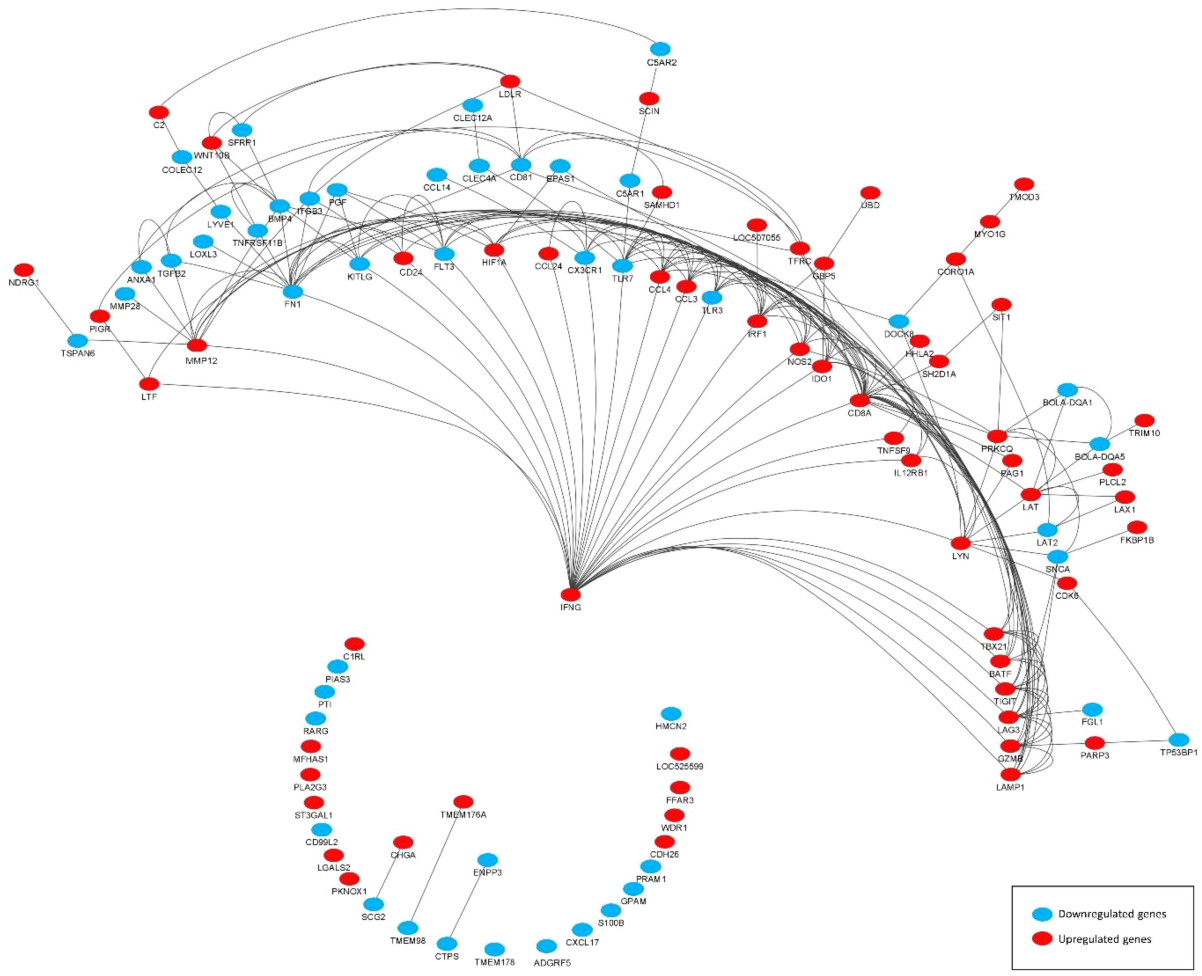
Gene interaction network representing the functional relationships between differentially expressed genes (DEGs) (subset of immune related). The edges present a physical interaction between DEGs, either through their genes’ products, such as proteins, or when one gene regulates the expression of another.

DEGs associated with immunity (termed immune-related DEGs) were selected based on the UniProt immune system process database and were further used for GO analysis (**Supplementary S3**). Immune-related DEGs along with their gene interaction network are shown in **Figure 4**. The majority of immune-related DEGs have physical interactions or exhibit coexpression or colocalization characteristics, indicating that they are activated or inhibited as a part of feedback signaling pathways in response to a stimulus.

**Figure 5:**
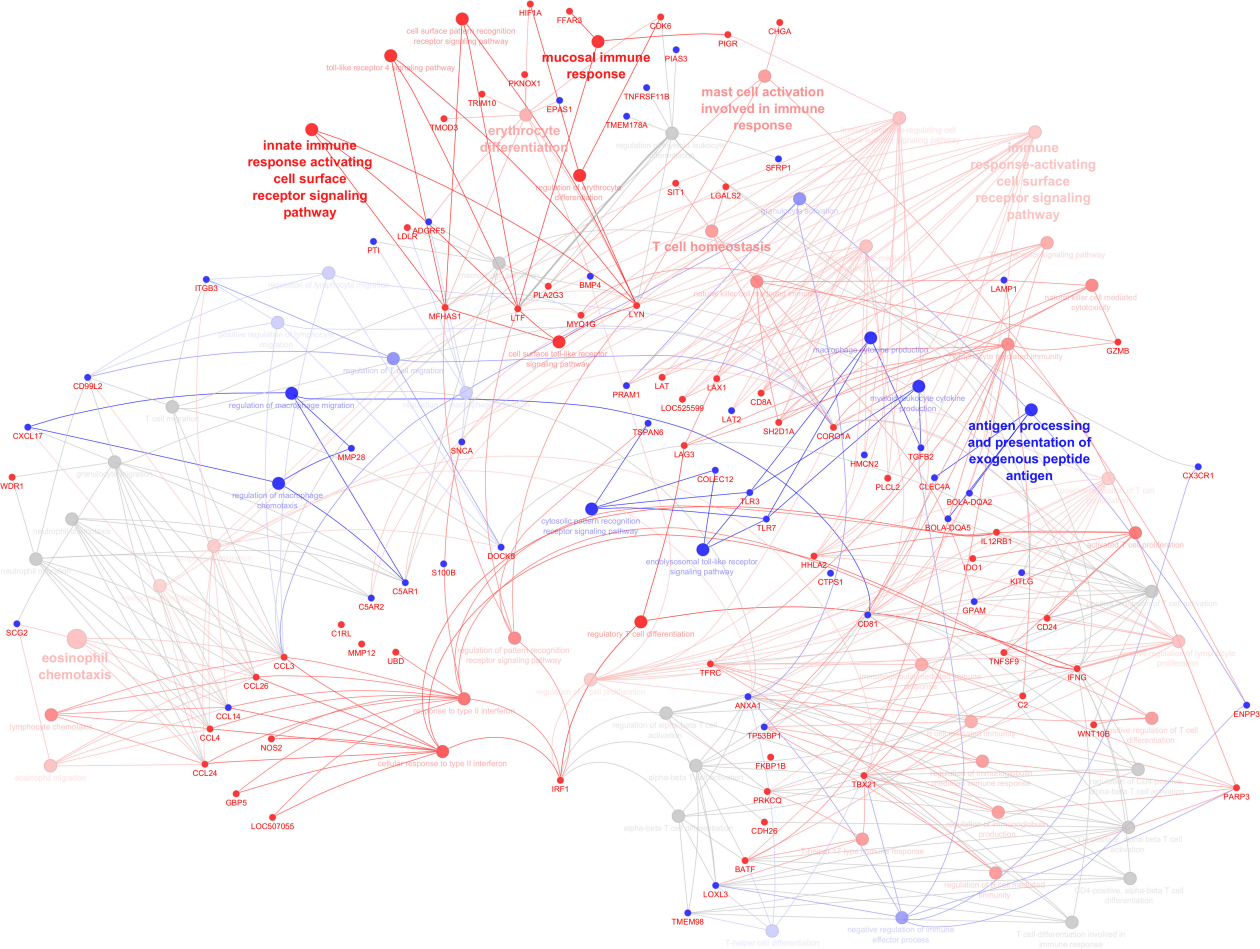
Illustration of immune system processes associated with immune-related genes in the infected calves. The network shows interconnections among different immune pathways. Pathways are clustered and represented by different colors indicating annotated to upregulated (red), annotated to downregulated (blue), or neutral annotated by both up and downregulated genes (grey). The hue intensity corresponds to q values (FDR). Genes that were initial for pathway enrichment or shared between pathways are highlighted (upregulated: red, downregulated: blue).

As shown by gene network and ontology analyses, a large number of DEGs are implicated in a very complex feedback and feedforward network of cells, cytokines, and signaling molecules, making understanding their system-level function challenging. Bioinformatic analysis has been used to classify the large DEG sets into their “biological process” (GO-BP) as well as holistically predict induced or inhibited immune pathways in response to DEG. **Figure 5** illustrates all immune pathways associated with upregulated or downregulated genes holistically. As a result, it was found that pathways related to the surface pattern recognition receptor (PRR) signaling pathway, mucosal immune response, TLR signaling pathway, antigen presenting cells, mast cells, eosinophils, NK cells, T cells, B cells, and Ig production were activated. In contrast, pathways related to neutrophils, macrophages, cytosolic pattern recognition, and cytokine production were annotated by downregulated genes. The full list of annotated pathways and their associated genes are provided in **Supplementary S4**.

## 4 Discussion

Studying neonatal calves’ early immune responses against *C. parvum* infection aims to identify key molecular players and pathways involved in establishing immunity, as well as discussing possible approaches adapted by protozoa to counteract. Immunohematological parameters on day 7 post infection were not significantly affected by *C. parvum* infection, despite visually different patterns between groups. However, we observed a significant impact of infection on the gene expression profile of the intestinal mucosa on day 7 of infection.

### 4.1 Innate immunity

Intestinal epithelial (and M) cells are the first cells that sense parasites and trigger signals which recruit specialized innate immune cells, such as NK cells, DCs, macrophages, mast cells (7), and consequently adaptive immune cells, such as T cells (26). We observed a downregulation of cytosolic PRR signaling markers including collectin-12 (COLEC12), TLR7, and tetraspanin 6 (TSPAN6). However, genes associated with the cell surface PRR, including lactotransferrin (LTF), tyrosine-protein kinase (LYN), and malignant fibrous histiocytoma-amplified sequence 1 isoform x1 (MFHAS1) were upregulated in the infected group. These upregulated genes are part of TLR4 signaling pathway, which recognize pathogen-associated molecular patterns on the cell surface as well as intracellular compartment membranes (e.g., endosomes) and the cytoplasm (e.g., in macrophages) (27). Previously, TLR2 and TLR4 have been implicated in host-cell responses during *C. parvum* infection of cultured bovine intestinal epithelial cells (28). Furthermore, previous studies have shown increased TLR4 expression (29) and activated TLR4-mediated response (30) during *C. parvum* infection in a mouse model.

A TLR signal in turn activates the pro-inflammatory transcription factors NF-κB, IRFs, such as IRF1, and mitogen-activated protein kinases to regulate the expression of cytokines, chemokines, and type I IFNs (6). In accordance, we observed an enrichment of the NF-κB signaling pathway by overexpression of protein kinase C theta (PRKCQ), an enzyme that directly or indirectly catalyzes IκB phosphorylation (31), and is thus crucial for NF-κB activation and signal transduction. NF-κB signaling is the central regulator of the inflammatory response to pathogens and a crucial ‘peace-keeping’ mechanism (32). In the canonical pathway, NF-κB signals drive the transcription of chemokines, acute phase proteins, cytokines, cell adhesion molecules, and components of the complement cascade in both the innate and adaptive immune response (33).

*C. parvum* infection has been shown to induce the release of chemokines, including CCL12, CCL20, CXCL1, CXCL8, CXCL10, and CX3CL1 that attract neutrophils, monocytes and DCs (34–38). In our study, several chemokines, including chemokine ligand CCL3 (also known as macrophage inflammatory protein 1-alpha), CCL4 (also known as macrophage inflammatory protein 1-beta), CCL24, CCL26, and CCL27 and chemokine receptors CXCR3 and CXCR6 were upregulated, whereas CCL14 and CXCL17 as well as chemokine receptors CX3CR1 were downregulated. Despite the fact that multiple chemokines have more than one receptor and vice versa, a number of them are specialized with a high degree of specificity to recruit a specific type of immune cells (39). Our knowledge of cattle chemokine families and their specificity is limited; the ones identified here were not previously reported in dairy cows with respect to *C. parvum* infection, and also differ from those commonly described in the other species, emphasizing the interspecies differences. As reported in the literature, during cryptosporidium infection, chemokines assist with the migration of inflammatory and adaptive immune cells such as neutrophils, NK cells, macrophages, and DCs to the infection site (40, 41). Our bioinformatics analysis suggests the possible recruitment of granulocyte (neutrophils, eosinophils, mast cells and basophils) and mononuclear cell (lymphocytes and monocytes) recruitment during calves’ *C. parvum* infection, which require further validation.

Upon recruitment, innate immune cells secrete pro-inflammatory and antimicrobial mediators including cytokines (IFN). Canonic IFN signaling pathway was observed to be essential for the development of effective immune responses against *C. parvum* (42). In this regard, we observed the overexpression of IFNG, IL-12 receptor beta 1 (IL12RB1), IRF-1, nitric oxide synthase 2 (NOS2), Ubiquitin D (UBD), guanylate binding protein 5 (GBP5), and guanylate-binding protein 4 (LOC507055) associated with response to type II IFN pathway. In accordance, a previous study found that IFN-γ protein abundance increased in neonatal calves infected with *C. parvum* (43). It is generally accepted that IFN-γ is the crucial defensive cytokine in intracellular protozoan parasite infection, although the contribution of type I and type III IFNs has also been reported recently, as reviewed by (42). Moreover, the IFN signaling pathway induces the GTPase family, including GBP5 and LOC507055, which are involved in many important cellular processes, inducing the activation of inflammasomes and innate immunity (44). Among the other secreted mediators, three identified upregulated genes - LTF, fatty acid receptor (FFAR)-3, and polymeric Ig receptor (PIGR) - were annotated to the mucosal immune response to strengthen and maintain intestinal mucosal immune homeostasis.

Several types of immune cells have been reported to produce IFN-γ during intracellular parasite infections; however, NK cells (45), and CD4+/CD8+ (double positive) T cells (46) are the most predominant sources. Infection with *C. parvum* has been reported to increase the number of neutrophils, eosinophils, NK cells, and T cells in peripheral blood of mice (47). Accordingly, we observed the annotation of upregulated genes to NK cells, mast cells, eosinophils, lymphocytes, and monocytes. It is now widely understood that NK cells play a major role in innate resistance to protozoa, mainly through the production of cytokines, but also by cytotoxic effects on infected epithelial cells (48). In the infected group, we observed enrichment of NK cell-mediated cytotoxicity associated with the upregulation of Coronin 1A (CORO1A), Granzyme B (GZMB), LAG3, SH2 domain–containing protein 1A (SH2D1A). In line, lambs infected with *C. parvum* have shown high activity and participation of CR1+/CD16+ and NCR1+/CD16- NK cells in the gut (8). We found an upregulation the two potent cytotoxic molecules CORO1A (49) and GZMB (50) that are utilized by NK cells and cytotoxic T lymphocytes to phagocyte infected cells opsonized by antibodies. In accordance, the expression of CD160, Fc Gamma Receptor IIIa (FCGR3A), GZMB, and CCL3 are shown to be upregulated in mature NK cells (51).

The cells recruited next, DCs, play a crucial role in preventing *C. parvum* infection by secreting cytokines and type I IFNs via TLR4 receptor activation (52). Weak production of chemokines (CCL3, CCL4, CCL5, CCL22, CXCL9, and CXCL10) by intestinal epithelial cells led to fewer intestinal CD103+ DCs in neonatal mice (as compared with adults). However, acute *C. parvum* infection induced the production of CXCR3-binding chemokines (by intestinal epithelial cells), and facilitated the recruitment of CD103+ DCs (36). In our study, the expression of several DC differentiation-related genes including Ubiquitin D (UBD), Src family tyrosine kinase (LYN), and B cell activating transcription factor (BATF) was upregulated, suggesting that DCs are activated and probably turned to mature antigen-presenting cells upon differentiation. Mature DCs serve as a bridge between the innate and adaptive immune system that recognize and present antigenic peptides (MHC class II) to naive CD4 and CD8 T cells. The mature DC produces cytokines, such as IL-12, and expresses chemokine receptors, which promotes the differentiation of naïve CD4 T cells into type 1 T helper (Th1) (53). In line with that, we observed the overexpression of interleukin-12 receptor beta 1 (IL12RB1), a type I transmembrane receptor essential to IL12 activation.

### 4.2 Adaptive immune response

The neonatal calves’ adaptive immune system was not completely established, but this does not mean that the system is not responsive to infection; in fact, the response is initiated but is underdeveloped. We identified the enrichment of several pathways related to T cell activation, migration, proliferation, differentiation, and signaling pathways indicating their importance. In accordance, CD4+ and CD8+ T cells are shown to be essential immune mediators in response to cryptosporidiosis and in its recovery in calves (54, 55). In addition, a higher number of CD8+ T cells was reported in neonatal lamb gut tissue after infection with *C. parvum* (8), which act to lyse infected intestinal epithelial cells to reduce parasite burdens.

In our study, the T cell receptor (TCR) signaling pathway is activated as a result of presenting antigens by DCs or B cells. The TCR pathway was annotated by overexpression of CD8 subunit alpha (CD8A), CD8 subunit beta (CD8B), Herv-H long terminal associating 2 (HHLA2), LOC525599, PRKCQ, LAT and SH2D1A, and downregulation of CD81 and PML-RARA regulated adaptor molecule 1 (PRAM1). A number of those immune related DEGs are surface costimulatory (HHLA2, CD24, CD81, TNFSF9) or co-inhibitory (LAG3) markers, mainly belong to the B7 or TNF family and their balance ensures that T cells are activated and respond properly (56, 57). The TCR activates CD4+ as well as CD8+ T cells, resulting in clonal expansion and differentiation of T cells into effector cells (Th1 and Th2 type). Several genes were annotated to T cell activation, migration, and differentiation. In particular, we observed the upregulation of genes involved in the Th1, Th2, and Th17 cells differentiation including IL12RB1, PRKCQ, and T-box transcription factor 21 (TBX21). Th1 subsets of CD4+ T cells are involved in cell-mediated immune responses by producing cytokines such as IFNG and TNF, whereas the Th2 subset mediates humoral immune responses by producing cytokines that promote the proliferation and differentiation of B cells. Th17 cells are the first subset of CD4+ Th cells to differentiate in response to antigen presenting cells (58). Th17 cells produce IL-17, and contribute to host defense against pathogens at mucosal sites (59) by a chemotactic effect on neutrophils (58). PRKCQ, a member of the PKC family and one of the essential molecules within T cells, was recently shown to be a nuclear transcription factor required for the activation of Th2 and Th17 cells (60).

The other component of adaptive immune cells consists of B cells, whose involvement in the immune response during *Cryptosporidium* infection is controversial. It has been reported that B cell free mice are able to remove *C. parvum* parasites, and that Igs do not control protozoan parasite replication (41). However, it is known that individuals with deficiencies in Igs (IgM and IgA) are more susceptible to *Cryptosporidium* infection (41). Moreover, cytokines and chemokines produced by B cells directly affect DCs and T cells. Therefore, B cells seem to play an important role during parasite infection, but this role is not only protective but also pathologic as reviewed by Amezcua Vesely et al. (61). NGS results revealed an enrichment of pathways associated with B cell activation, differentiation and migration, as well as Ig production. It is noteworthy that protozoan parasites have evolved protective mechanisms, which influence the development of different compartments of B cells and their survival by causing apoptosis. Downregulated genes were CD81, CTP Synthase 1 (CTPS1), CD antigen 135 (CD135, also known as FLT3), hemicentin 2 (HMCN2), linker for activation of T cells family member 2 (LAT2), secreted frizzled related protein 1 (SFRP1), tumor protein P53 binding protein 1 (TP53BP1), and upregulated markers were BATF, IFNG, lymphocyte transmembrane adaptor 1 (LAX1), poly(ADP-ribose) polymerase family member 3 (PARP3), phospholipase C Like 2 (PLCL2), ST3 beta-galactoside alpha-2,3-sialyltransferase 1 (ST3GAL1), transferrin receptor (TFRC), LYN, and TBX21.

Several of these genes encode cell surface receptors that are induced (costimulatory) or downregulated (coinhibitory) during various stages of B cell activation or proliferation. For example, the Class III receptor tyrosine kinase, FLT3, is another membrane-bound cell surface receptor expressed on B-cell progenitors and plays a crucial role in B cell development, though it is downregulated during early B cell differentiation and upregulated on activated germinal center B cells (62). NTAL/LAB/LAT2 (linker for activation of B cells, T cells, and non-T cells) is another transmembrane protein mainly expressed in spleen and hematopoietic cells, including B cells, mast cells, NK cells, and monocytes, which becomes downregulated during the development of B cells (63). BATF is induced upon CD4+ Th cell activation and induces Th17 cytokine production (64) and antibody class switching in B cells (65). TFRC is also a transmembrane glycoprotein required for iron uptake in cells, which is expressed less in resting B cells, but increases during their activation as iron promotes proliferation of B cells (66). Additionally, a number of Ig-producing related genes, including PARP3, TBX21, and TFRC, were upregulated in the infected group. TBX21 is a transcription factor which has been linked to the transformation of Th1 cells, thus regulating the balance of Th1/Th2 cells and controlling the production of IFN-γ (67). Moreover, it has been reported that TBX21 could regulate B cell Ig production via promoting the production of IgG2a, IgG2b, and IgG3 and inhibiting the production of IgG1 and IgE (68). Parp3 plays an immunoglobulin class switch recombination (CSR)-specific role which allows B cells to switch antibody classes (from IgM to IgA, IgG or IgE) during infections in order to establish highly specific and pathogen-adapted antibody responses to pathogens (69).

As a result of protozoan infection, the intestinal integrity and immune system were compromised, making the calves susceptible to secondary infections with bacteria, coronavirus, and rotaviruses. The enrichment of specific pathways directly or indirectly related to bacterial (or other microorganisms’) infections suggests co-occurring infection, though further investigation is required to confirm it. To the best of our knowledge, there hasn’t yet been a published result on vaccine discovery for NCD. It has only been recently that the *C. parvum* gp40/15 protein has been suggested as a promising vaccine candidate (70), which is based upon some ongoing attempts that are being made to resurrect hope for the vaccine development. To compare the upcoming results of potential vaccines against *C. parvum*, this study provided a comprehensive database of immune markers and their expression based on an *in vivo* experiment.

## 5 Conclusion

Developing effective prevention measures and therapies requires a deep understanding of the responses triggered by *C. parvum* in the gut mucosa during early stages of parasite-host interactions. In this study, whole transcriptome sequencing and subsequent bioinformatics analysis provided a deeper understanding of cellular and molecular interactions occurring in the gut mucosa during *C. parvum* invasion. This includes assessment of novel and detailed feedback and feedforward regulatory mechanisms establishing the crosstalk between innate and adaptive immune response in neonate calves, highlighting the importance of specific cytokines, chemokines, and receptors within specialized innate and adaptive immune cells and possible interference triggered by parasites. Even though the enrichment of specific adaptive immune pathways was surprising, it appears that the induced mechanisms were not able to mount an adequate (mature) immune response to protect against *C. parvum* and consequently restore immune homeostasis. Due to delayed adaptive immunity response and the possibility of secondary co-infections, future studies will likely focus on other strategies beside or complementary to vaccines for alleviating diarrhea, such as improving gut microbiota against pathogens or regulating absorption and secretion mechanisms.

## Data availability statement

RNA-Seq datasets are submitted to the ENA repository (https://www.ebi.ac.uk/ena, Project number PRJEB70797, accession numbers ERR12331848 - ERR12331857) at EMBL-EBI.

## Ethics statement

The animal study was approved by the German animal protection legislation and was licensed by the Landesdirektion Leipzig, Germany as TVV 19/20. The study was conducted in accordance with the local legislation and institutional requirements.

## Author contributions

AV: Formal Analysis, Visualization, Writing – original draft, Writing – review & editing. FD: Conceptualization, Data curation, Investigation, Project administration, Writing – review & editing. LB: Data curation, Methodology, Project administration, Resources, Writing – review & editing. WL: Data curation, Investigation, Methodology, Writing – review & editing. CH: Data curation, Investigation, Methodology, Writing – review & editing. RU: Data curation, Investigation, Methodology, Writing – review & editing. CD: Conceptualization, Data curation, Investigation, Methodology, Writing – review & editing. CK: Methodology, Resources, Software, Writing – review & editing. HH: Conceptualization, Funding acquisition, Project administration, Supervision, Writing – review & editing.
